# Supplementary material for: Integrating human endogenous retroviruses into transcriptome-wide association studies highlights novel risk factors for major psychiatric conditions
Source: Nat Commun. 2024 May 22;15:3803. doi: 10.1038/s41467-024-48153-z (PMC11111684; doi:10.1038/s41467-024-48153-z)
Supplement: Supplementary file 1 — Supplementary Information [file 41467_2024_48153_MOESM1_ESM.pdf]

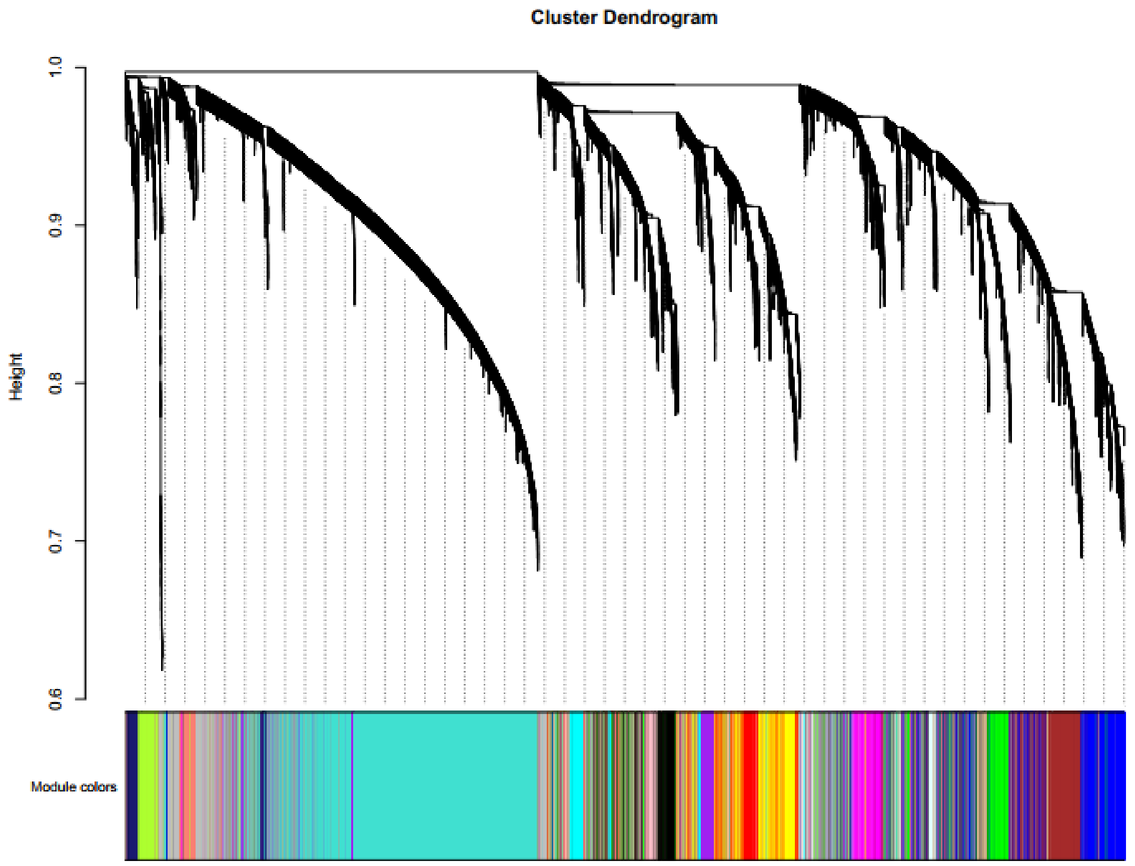

Supplementary Figure 1. Clustering dendrogram of the expression network identified in the dorsolateral prefrontal cortex. Expression dissimilarity, demonstrated as height in the Y axis, was calculated based on topological overlap using WGCNA. The assigned module colours are shown below (N = 563 biologically independent samples).
