# Supplementary material for: Integrating human endogenous retroviruses into transcriptome-wide association studies highlights novel risk factors for major psychiatric conditions
Source: Nat Commun. 2024 May 22;15:3803. doi: 10.1038/s41467-024-48153-z (PMC11111684; doi:10.1038/s41467-024-48153-z)
Supplement: Supplementary file 3 — Description of Additional Supplementary Files [file 41467_2024_48153_MOESM3_ESM.pdf]

Description of Additional Supplementary Files

File Name: Supplementary Data 1

Description: List of significant expression signatures associated with attention deficit hyperactivity disorder (GWAS N = 55,374), autism spectrum conditions (GWAS N = 46,350), bipolar disorder (GWAS N = 413,466), major depressive disorder (GWAS N = 807,553), and schizophrenia (GWAS N = 161,405) in an rTWAS analysis. The table only shows expression signatures that survive multiple testing correction for the number of expressed features in the dorsolateral prefrontal cortex using the Bonferroni method (two-sided P value cut-off = 6.10 x 10e-6), with HERV IDs highlighted in red.

File Name: Supplementary Data 2

Description: Conditional and joint analyses performed within the FUSION pipeline further support HERV expression features as implicated in major psychiatric disorders. HERVs (highlighted in red) and canonical genes within this table correspond to genetic features whose expression-trait associations are conditional on the observed GWAS statistics at their loci.

File Name: Supplementary Data 3

Description: Fine-mapping analysis performed within the FOCUS pipeline further support specific HERV expression features as likely to explain GWAS signals at their locations. The table shows only expression features with a posterior inclusion probability (PIP) greater than 0.5 (i.e., more likely to explain GWAS signal).

File Name: Supplementary Data 4

Description: Module assignment for HERVs (highlighted in red) and genes expressed in the adult dorsolateral prefrontal cortex, according to a weighted correlation network analysis (N = 563 biologically independent samples).

File Name: Supplementary Data 5

Description: Gene ontology analysis from WGCNA co-expression modules. The table shows the top 10 Bonferroni-significant GO terms enriched per module (Bonferroni adjusted P < 0.05).
