# Supplementary material for: Integrating human endogenous retroviruses into transcriptome-wide association studies highlights novel risk factors for major psychiatric conditions
Source: Nat Commun. 2024 May 22;15:3803. doi: 10.1038/s41467-024-48153-z (PMC11111684; doi:10.1038/s41467-024-48153-z)
Supplement: Supplementary file 9 — Reporting Summary [file 41467_2024_48153_MOESM9_ESM.pdf]

nature portfolio

Corresponding author(s): Rodrigo R. R. Duarte, Timothy R. Powell

Last updated by author(s): Mar 10, 2024

Reporting Summary

Nature Portfolio wishes to improve the reproducibility of the work that we publish. This form provides structure for consistency and transparency in reporting. For further information on Nature Portfolio policies, see our Editorial Policies and the Editorial Policy Checklist.

Statistics

For all statistical analyses, confirm that the following items are present in the figure legend, table legend, main text, or Methods section.

n/a	Confirmed	
☐	☒	The exact sample size (*n*) for each experimental group/condition, given as a discrete number and unit of measurement
☐	☒	A statement on whether measurements were taken from distinct samples or whether the same sample was measured repeatedly
☐	☒	The statistical test(s) used AND whether they are one- or two-sided<br>*Only common tests should be described solely by name; describe more complex techniques in the Methods section.*
☐	☒	A description of all covariates tested
☐	☒	A description of any assumptions or corrections, such as tests of normality and adjustment for multiple comparisons
☐	☒	A full description of the statistical parameters including central tendency (e.g. means) or other basic estimates (e.g. regression coefficient) AND variation (e.g. standard deviation) or associated estimates of uncertainty (e.g. confidence intervals)
☐	☒	For null hypothesis testing, the test statistic (e.g. *F*, *t*, *r*) with confidence intervals, effect sizes, degrees of freedom and *P* value noted<br>*Give P values as exact values whenever suitable.*
☒	☐	For Bayesian analysis, information on the choice of priors and Markov chain Monte Carlo settings
☒	☐	For hierarchical and complex designs, identification of the appropriate level for tests and full reporting of outcomes
☐	☒	Estimates of effect sizes (e.g. Cohen's *d*, Pearson's *r*), indicating how they were calculated

Our web collection on statistics for biologists contains articles on many of the points above.

Software and code

Policy information about availability of computer code

Data collection	N/A - our team was not involved in data collection. We obtained access to the CommonMind Consortium data via a formal application to the National Institute of Mental Health (NIMH) Repository and Genomics Resources (NRGR).
Data analysis	PLINK 1.9 (Chang et al., 2015)<br>bcftools 1.9 (Danecek et al., 2021)<br>Michigan Imputation Server 1.7.4 (Das et al., 2016)<br>samtools 1.5 (Li et al., 2009)<br>Picard 3.1.1 (Broad Institute, 2022)<br>Trimmomatic 0.38 (Bolger et al., 2014)<br>Bowtie2 2.3.5.1 (Langmead and Salzberg, 2012)<br>Telescope 1.0.2 (Bendall et al., 2019)<br>kallisto 0.44.0 (Bray et al., 2016)<br>biomaRt 2.42.0 (Durinck et al., 2009)<br>tximport 1.14.0 (Soneson et al., 2016)`<br>liftOver (single version) (UCSC)<br>limma 3.42.0 (Ritchie et al., 2015)<br>sva 3.34.0 (Leek et al., 2019)<br>FUSION (single version as of Mar 2024) (Gusev et al., 2016)<br>FOCUS (single version as of Mar 2024) (Mancuso et al., 2019)<br>WGCNA 1.69 (Langfelder and Horvath, 2008)<br>anRichment 1.22 (Langfelder, 2019)

Homer 4.11 (Heinz et al., 2010)
Bash 5.0.17 (GNU Project Bourne Again SHell)
R 3.6.3 (The R Project for Statistical Computing, Vienna, Austria)
Code used in this manuscript (single version) (FigShare repository, DOI: 10.18742/22179655)

For manuscripts utilizing custom algorithms or software that are central to the research but not yet described in published literature, software must be made available to editors and reviewers. We strongly encourage code deposition in a community repository (e.g. GitHub). See the Nature Portfolio guidelines for submitting code & software for further information.

Data

Policy information about availability of data

All manuscripts must include a data availability statement. This statement should provide the following information, where applicable:

- Accession codes, unique identifiers, or web links for publicly available datasets
- A description of any restrictions on data availability
- For clinical datasets or third party data, please ensure that the statement adheres to our policy

The RNA-sequencing and genotype data from the CommonMind Consortium cohort are available under restricted access for containing sensitive data. Access can be obtained via an application to the NIMH Repository and Genomics Resource (NRGR) (https://www.synapse.org/#!Synapse:syn2759792/). GWAS summary statistics were downloaded from the Psychiatric Genomics Consortium website (https://pgc.unc.edu/for-researchers/download-results/). SNP weights derived from our analyses and example reference panels are freely available from a FigShare repository (DOI: 10.18742/22179655; https://doi.org/10.18742/22179655).

Research involving human participants, their data, or biological material

Policy information about studies with human participants or human data. See also policy information about sex, gender (identity/presentation), and sexual orientation and race, ethnicity and racism.

Reporting on sex and gender	Sex was previously identified as a source of variation in terms of expression of HERVs and canonical genes (see Duarte et al 2019; Hoffman et al 2019). Thus, we included it as a covariate in our analyses and/or adjusted the data accordingly. We opted not to stratify our rTWAS analysis by sex, given the underlying assumption of the TWAS methodology that genetic influences on gene expression remain consistent across sexes. This decision is particularly relevant as our study focuses solely on autosomal genetic features due to current limitations in investigating sex chromosome-related factors within the field.
Reporting on race, ethnicity, or other socially relevant groupings	We provide the self-reported ancestry of the cohort for reference purposes. In our analyses, we infer ancestry (African or European) from the samples using genetic data, and use this information to split the cohort into two sub-samples. This is necessary because genetic structure differences between the two populations can influence our analyses.
Population characteristics	We identified 563 individuals of European ancestry, including 242 unaffected individuals, 223 individuals diagnosed with schizophrenia, 91 with bipolar disorder, and 7 broadly diagnosed with an affective disorder. Besides the 27 individuals who were >90 years old, the remaining individuals (N = 536) were on average 58.85 years old at the time of death (standard deviation (SD) = 18.78; range = 17 – 90). The cohort consisted of 196 females (35%) and 367 males (65%). The mean post-mortem interval was 20.99 hours (SD = 12.87, range = 2.00 – 84.50) and the mean RNA integrity number was 7.60 (SD = 0.91, range = 4.60 – 9.60).<br><br>We also identified 229 individuals of African ancestry, which consisted of 139 unaffected individuals, 80 individuals diagnosed with schizophrenia, 9 with bipolar disorder, and 1 broadly diagnosed with an affective disorder. Besides the 7 individuals who were >90 years old, the remaining individuals (N = 222) were on average 49.45 years old at the time of death (standard deviation (SD) = 17.41; range = 17 – 89). The cohort consisted of 93 females (41%) and 136 males (59%). The mean post-mortem interval was 28.04 hours (SD = 12.67, range = 1.60 – 168.00) and the mean RNA integrity number was 7.65 (SD = 0.88, range = 5.60 – 9.30).
Recruitment	n/a - Our group was not involved in patient recruitment. The data was obtained via a formal application for access to the CommonMind Consortium dataset, which is safeguarded by the NIMH NRGR, as described in the Data Availability statement.
Ethics oversight	n/a - The study protocols were approved by the institutional review board at each centre involved with recruitment and data sharing. Informed consent and permission to share the data were obtained from all individuals, in compliance with the guidelines specified by the institutional review boards of the recruiting centres.

Note that full information on the approval of the study protocol must also be provided in the manuscript.

Field-specific reporting

Please select the one below that is the best fit for your research. If you are not sure, read the appropriate sections before making your selection.

☒ Life sciences ☐ Behavioural & social sciences ☐ Ecological, evolutionary & environmental sciences

For a reference copy of the document with all sections, see nature.com/documents/nr-reporting-summary-flat.pdf

Life sciences study design

All studies must disclose on these points even when the disclosure is negative.

Sample size	The initial sample size was 910 independent samples (i.e., post-mortem dorsolateral prefrontal cortex samples from the CommonMind Consortium cohort, for whom genetic and RNA-seq data were available). We selected individuals of European (N = 563) and African (N = 229) ancestry to construct the SNP weights, using the 1,000 Genomes Phase 3 as reference panel.
Data exclusions	Once individuals of European or African ancestry were selected, we applied standard quality control filtering criteria in relation to genotype data processing (e.g., we removed samples with excess heterozygosity (mean heterozygosity rate above 3 standard deviations), high likelihood of relatedness (pihat > 0.2), those with missing genotype information > 0.05, or with mismatched sex information). Principal component analysis of the expression data was employed for visual inspection to identify and subsequently remove obvious outliers.
Replication	To assess the reproducibility of our work concerning canonical genes, we leveraged insights from previous schizophrenia TWASs. For instance, we replicated schizophrenia expression signatures identified previously in a TWAS that considered cis-heritable expression in a subset of the CMC cohort (e.g., genes NAGA, Z = 7.74, P = 9.58 x 10e-15; SNAP91, Z = 4.80, P = 1.61 x 10e-6; TAOK2, Z = -7.44, P = 1.04 x 10e-13) (Gusev et al., 2018). We also replicated schizophrenia TWAS signatures identified previously in a study that considered cis-heritable expression in the developing human brain (e.g., genes SF3B1, Z = 6.99, P = 2.78 x 10e-12; MAPK3, Z = 5.68, P = 1.39 x10e-8; FURIN, Z = -8.44, P = 3.11 x 10e-17) (Hall et al., 2020).
Randomization	n/a - In a TWAS, expression data is analysed according to genotype (e.g., AA/Aa/aa) at each single nucleotide polymorphism, and this information is tested against results from GWAS, which is considered a hypothesis-free approach. We controlled expression for covariates that were previously identified as having an impact on gene/HERV expression, such as age, sex, post-mortem interval, RNA integrity number, and institution of sample origin (see Duarte et al., 2019; Hoffman et al., 2019).
Blinding	n/a - In a TWAS, which is a hypothesis-free approach, blinding is not applicable during data collection or analysis, as there are no predetermined groups or hypotheses to be blinded to.

Reporting for specific materials, systems and methods

We require information from authors about some types of materials, experimental systems and methods used in many studies. Here, indicate whether each material, system or method listed is relevant to your study. If you are not sure if a list item applies to your research, read the appropriate section before selecting a response.

Materials & experimental systems

n/a	Involved in the study
☒	☐ Antibodies
☒	☐ Eukaryotic cell lines
☒	☐ Palaeontology and archaeology
☒	☐ Animals and other organisms
☒	☐ Clinical data
☒	☐ Dual use research of concern
☒	☐ Plants

Methods

n/a	Involved in the study
☒	☐ ChIP-seq
☒	☐ Flow cytometry
☒	☐ MRI-based neuroimaging

Plants

Seed stocks	*Report on the source of all seed stocks or other plant material used. If applicable, state the seed stock centre and catalogue number. If plant specimens were collected from the field, describe the collection location, date and sampling procedures.*
Novel plant genotypes	*Describe the methods by which all novel plant genotypes were produced. This includes those generated by transgenic approaches, gene editing, chemical/radiation-based mutagenesis and hybridization. For transgenic lines, describe the transformation method, the number of independent lines analyzed and the generation upon which experiments were performed. For gene-edited lines, describe the editor used, the endogenous sequence targeted for editing, the targeting guide RNA sequence (if applicable) and how the editor was applied.*
Authentication	*Describe any authentication procedures for each seed stock used or novel genotype generated. Describe any experiments used to assess the effect of a mutation and, where applicable, how potential secondary effects (e.g. second site T-DNA insertions, mosiacism, off-target gene editing) were examined.*
